# M. Piorry on the Efficacy of Injections of Infusion of Cubebs in Vaginitis

**Published:** 1842-11-19

**Authors:** 


*On the Efficacy of Injections of Infusion of Cubebs in Vaginitis.* By M. Piorry.—A woman, 23 years of age, had suffered for nine months from an intense *urethro-vaginitis*, attended with acute pain, and an abundant discharge. She had tried every usual remedy for its removal without success. M. Piorry ordered an injection made with one ounce of cubebs powder, infused in one pound of water, to be thrown into the vagina six times daily. In two days the discharge had greatly diminished; but the inflammation in the urethra remained intense as before. She was therefore ordered to take 45 grains of powder of cubebs every hour, and in twelve days the pain and discharge had entirely left her, and she was dismissed cured.—*Ibid, from Gazette des Hôpitaux*, May, 1842.

